# Immunotherapy-induced pneumonitis: cases report

**DOI:** 10.1590/S1679-45082018RC4030

**Published:** 2018-06-15

**Authors:** Henrique Alkalay Helber, Aline Lury Hada, Raquel Baptista Pio, Pedro Henrique Zavarize de Moraes, Diogo Bugano Diniz Gomes

**Affiliations:** 1 Hospital Israelita Albert Einstein, São Paulo, SP, Brazil

**Keywords:** Pneumonitis/diagnosis, Immunotherapy, AntiPD1/AntiPDL1, Adrenal cortex hormones/adverse effects, Case reports, Pneumonite/diagnóstico, Imunoterapia, AntiPD1/AntiPDL1, Corticosteroide/efeitos adversos, Relatos de casos

## Abstract

Immunotherapy-induced pneumonitis is a rare complication with incidence estimated around 3%. This disease is difficult to diagnose and has great morbidity. For this reason, it became a challenge for oncologists and emergencists. We reviewed the case of five patients who used anti-PD1 (program cell death receptor antagonist 1) for antineoplastic treatment and developed treatment-induced pneumonitis. All patients had respiratory problems because of immunotherapy and presence of ground-glass radiologic change. Among all patients, only one had grade 5 pneumonitis, and delaying to begin corticosteroid therapy and worsening in clinical picture led to patient death. Other four patients with symptomatic grade 2 pneumonitis underwent corticosteroid therapy and had improvement in clinical and radiologic picture. Two patients were treated after an episode of pneumonitis, and no new pulmonary complications were observed until the end of this study. Immunotherapy-induced pneumonitis, although uncommon, can be potentially fatal. Medical team has the responsibility to pay attention for most common symptoms of the disease such as cough and dyspnea and conduct an early diagnosis and effective early treatment with corticosteroids.

## RESUMO

A pneumonite secundária à imunoterapia é uma complicação rara, com incidência estimada em cerca de 3%. No entanto, trata-se de uma intercorrência de difícil diagnóstico e com grande morbidade, que tem se tornado um desafio para oncologistas e emergencistas. Foram revisados os casos de cinco pacientes que fizeram uso de anti-PD1 (*program cell death receptor antagonist 1*) para tratamento antineoplásico e que evoluíram com quadro de pneumonite induzida pelo tratamento. Todos os pacientes apresentaram sintomas respiratórios em vigência de tratamento, com imunoterapia e presença de alteração radiológica em vidro fosco. Dentre estes pacientes, apenas um apresentou pneumonite grau 5, com atraso na introdução de corticoidoterapia, indo a óbito em decorrência do quadro. Os outros quatro pacientes apresentaram pneumonite grau 2, sintomática, sendo tratados com corticoidoterapia e evoluindo com melhora clínica e radiológica. Dois pacientes mantiveram o tratamento após o episódio de pneumonite, sem novas complicações pulmonares posteriores, até o momento. A pneumonite induzida por imunoterapia, apesar de ser um evento pouco frequente, pode acarretar grande morbidade, além de ser potencialmente fatal, cabendo à equipe médica ter atenção aos sintomas mais comuns, como tosse e dispneia, para diagnóstico precoce e tratamento efetivo, com uso precoce de corticoide.

## INTRODUCTION

Immunotherapy has shown important improvements for treatment of patients with advanced cancer. Programmed cell death protein-1 (PD-1) is a transmembrane T-cell receptor inhibitor. Pembrolizumabe and nivolumabe are monoclonal antibodies that ligate PD-1 receptor and block receptor activation and stimulate lymphocytes activity against tumor cells. Currently in Brazil, pembrolizumabe and nivolumabe are approved for treatment of melanoma, non-small cell lung cancer and renal cancer. However, some studies show promising results with these antibodies to treat several types of solid tumors. Studies including immunotherapy drugs suggest a favorable safety profile with immune-related adverse events that are often transitory, however, sometimes, severe. Among severe effects are diarrhea, skin changes, hepatitis and endocrinopathies. Pneumonitis is a little frequent complication with incidence estimated around 4%. However, this intercurrence is difficult to be diagnosed and present high morbidity.^(^ ^1^ ^)^

From June 2015 to November 2016 we reviewed medical records of 69 patients who were treated with anti-PD1 drugs. Of these, 5 developed pneumonitis at *Hospital Israelita Albert Einstein* (HIAE). This study describes diagnosis and management of these cases, and includes a brief literature review of the subject.

## CASE REPORT

### Case 1

**Figure 1:**
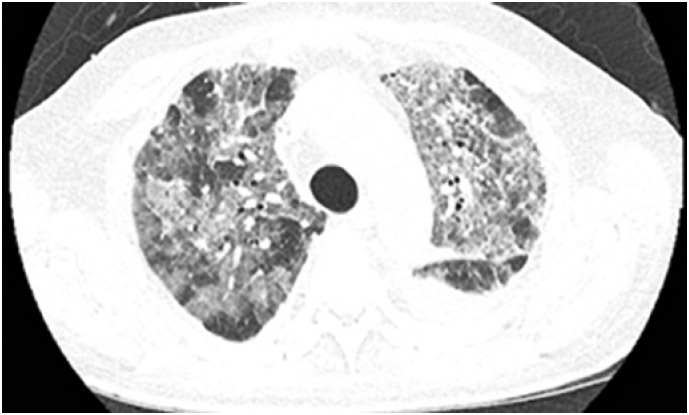
Diffuse bilateral ground-glass infiltrate, suggesting drug reaction

This was a 69-year-old man diagnosed with metastatic colorectal adenocarcinoma to liver, lung, and skeletal. He underwent previous treatments with schemes based on fluoropyrimidine, platinum, antiangiogenics, and irinotecan and cetuximab, however, the patient's disease had progressed with all these therapies. Treatment was initiated with pembrolizumabe 10mg/kg every 2 weeks, although this medicines use to the patient's disease is not approved in Brazil. After second administration, the patient reported fatigue and dyspnea. Upon physical examination, he had saturation of 83% in an open environment. Chest tomography evidenced infiltrated interstitial to left and bilateral pleural effusion without signs of pulmonary thromboembolism. Blood count showed leukocytosis with 21,580 leukocytes, 69% of them were segmented, 11% rod cells and 3% metamyelocyte. After thoracocentesis, antibiotic therapy with ceftriaxone and clarithromycin was initiated, and oxygen intake with nasal catheter was maintained, however, no improvement was observed. Reassessment of chest computed tomography showed increase of ground-glass infiltrate (Figure 1) that suggested drug reaction (acute interstitial pneumonitis pattern); a lung biopsy was not performed for histological confirmation. Because of worsening in patients’ conscious level and respiratory pattern, after discussion with his family, the sedation was initiated for patient's comfort.

### Case 2

This was a 73-year-old man diagnosed with melanoma on his right thigh. He underwent resection and clinical follow-up. After 8 years, he had untreatable metastatic lung melanoma without mutations. The patient was treated with dacarbazine followed by ipilimumab, but disease had progressed. After, we opted to begin pembrolizumab 2mg/kg administration every 3 weeks.

**Figure 2:**
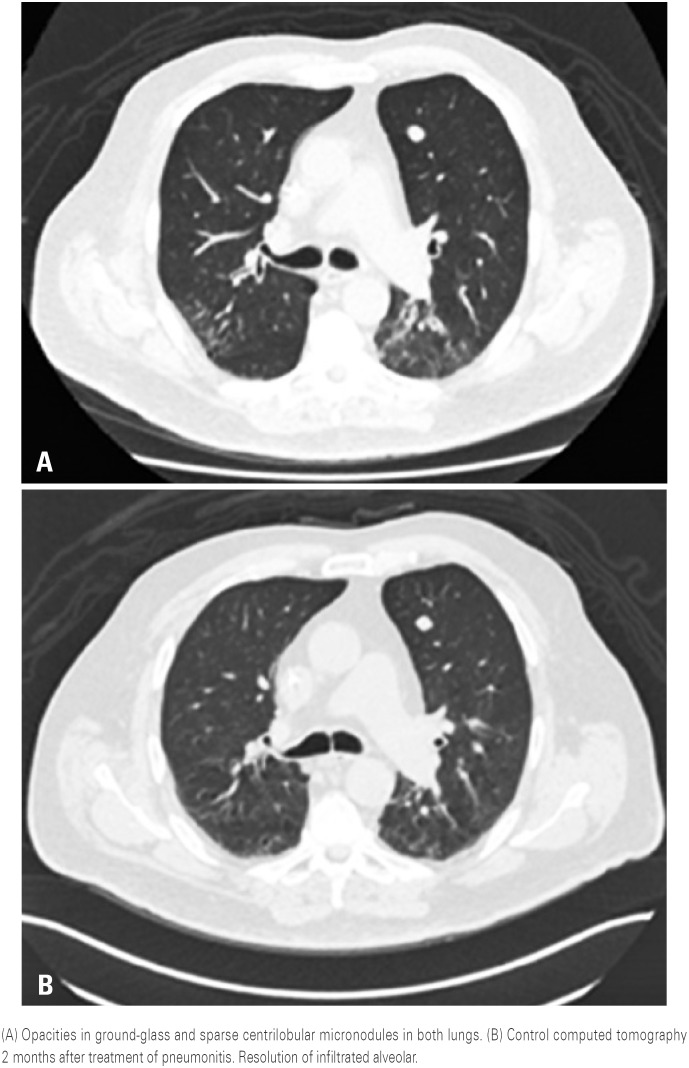
Computed tomography scan of a patient with immunotherapy-induced pneumonitis

Fourteen days after first cycle, the patient had a dry cough but without fever or other symptoms, no changes in blood count was observed. Chest computed tomography showed opacities in ground-glass in both lungs (Figure 2). The hypothesis raised was pembrolizumab-induced pneumonitis, although lung biopsy was not performed for histological confirmation. A treatment with 1mg/kg prednisone associated with antibiotic therapy and the patient had a rapid and important improvement in symptoms. Two months later, a staging computed tomography showed complete resolution of clinical feature (Figure 2). Patient maintained treatment with pembrolizumab, and showed good tolerance.

### Case 3

**Figure 3:**
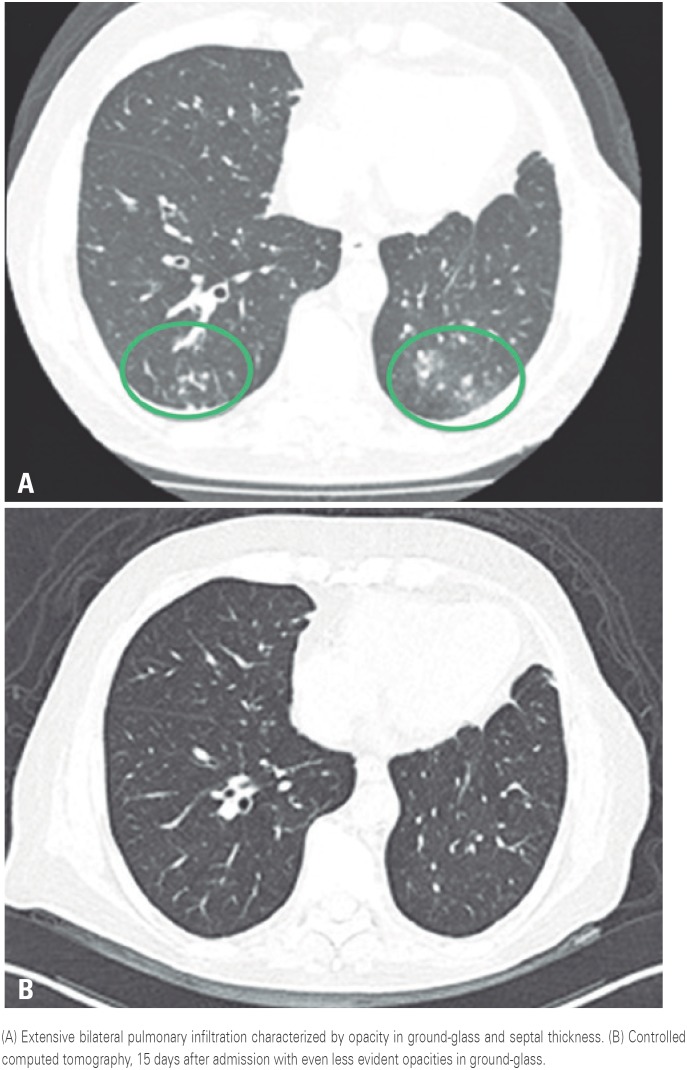
Computed tomography scan of a patient with immunotherapy-induced pneumonitis

This was an 81-year-old man diagnosed with untreatable stage IIIA lung adenocarcinoma without mutation. He underwent surgery followed by radiotherapy and adjuvant chemotherapy with carboplatin and pemetrexed. After 4 months of follow-up, the patient evolved with local recurrence. The affected site was irradiated but no response was seen, therefore, we opted for palliative chemotherapy with carboplatin and paclitaxel. A progression of the disease was also observed. Subsequently, we decided to begin immunotherapy with pembrolizumab 2mg/kg every 3 weeks. After four cycles, the patient had dyspnea and dry cough with oxygen saturation of 80%. Chest tomography showed extensive bilateral pulmonary infiltration (Figure 3), and blood count showed leukocytosis. No lung biopsy was performed to confirm pathology. Corticosteroid therapy was introduced with metilprednisolone 2mg/kg and antibiotic therapy. An important clinical improvement was seen and resolution of findings from controlled computed tomography (Figure 3).

### Case 4

This was a 54-year-old man diagnosed with pulmonary large-cell neuroendocrine carcinoma located and resected that evolved for metastatic disease. Initially the patient was treated with carboplatin and paclitaxel followed by cisplatin and etoposide, and radiotherapy for controlling specific injuries. The disease progressed to central nervous system and liver. We opted for immunotherapy with pembrolizumab 2mg/kg every 3 weeks.

**Figure 4:**
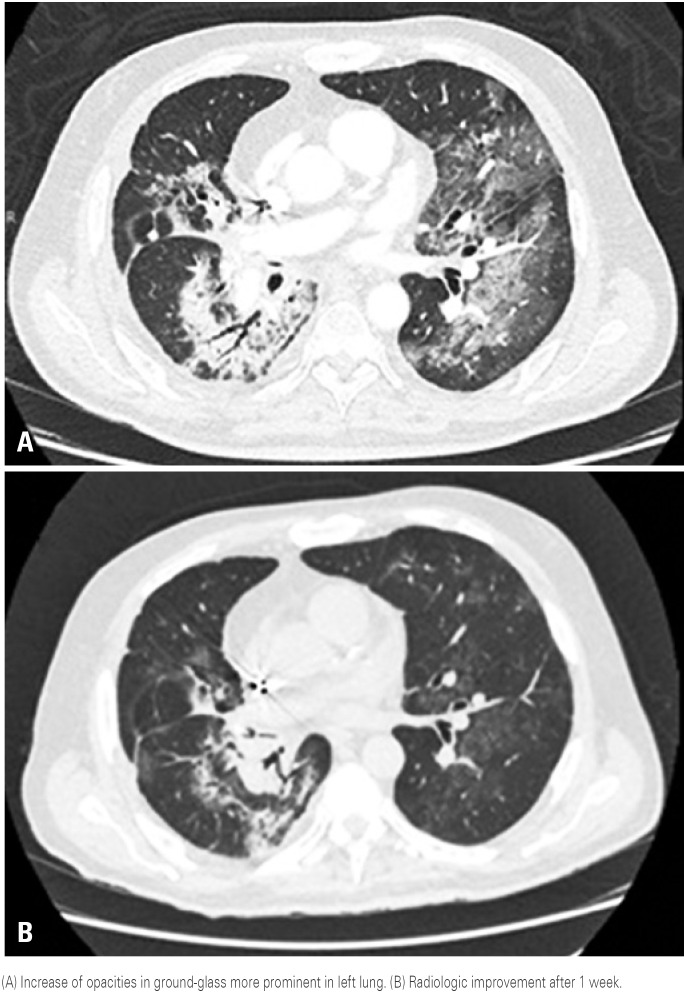
Computed tomography scan of a patient with immunotherapy-induced pneumonitis

After 5 cycles of treatment, patients’ clinical feature evolved with dyspnea and cough, but no fever. Upon clinical examination his oxygen saturation was 84% in an open environment. In thorax angiotomography the possibility of pulmonary thromboembolism was discarded and it identified opacities in bilateral ground-glass. Thus, we opted for treatment with metilprednisolone 2mg/kg associated with piperaciline-tazobactam 4.5g every 6 hours for the hypothesis of pneumonitis, although no histological confirmation was performed. An important clinical improvement was seen within 24 hours. The controlled computed tomography performed 1 week after treatment showed almost full resolution of pulmonary opacities (Figure 4).

### Case 5

This was a 70-year-old man with metastatic lung epidermoid carcinoma with multiple liver injuries. The first-line treatment adopted included nivolumab 3mg/kg every 2 weeks, even without these agents approval in Brazil and in the United States considering the patient's status because he had contraindication for platinum-based chemotherapy.

**Figure 5:**
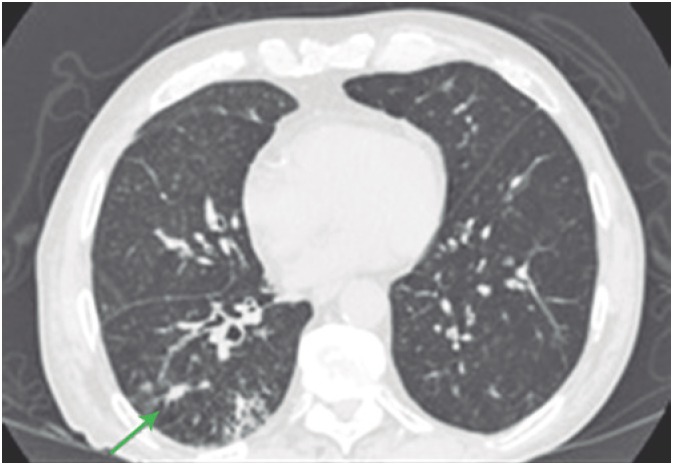
Nodular opacities in ground-glass, followed by increase of septal interstitial thickness

After 4 cycles, the patient had mental confusion, dyspnea and dry cough, without fever and oxygen saturation of 74% in an open environment. Blood count result was normal. We performed a chest tomography with appearance of infiltrated areas in ground-glass (Figure 5). The hypothesis raised was pembrolizumab-induced pneumonitis, although the lung biopsy was not performed to confirm the disease. We opted for treatment with methylprednisolone 60mg every 8 hours and also antibiotic therapy. The patient improved clinically within few hours and he was discharged asymptomatic after 3 days of hospitalization.

## DISCUSSION

We reviewed five cases of patients who developed immunotherapy-induced pneumonitis. Of these patients, only one had grade 5 pneumonitis and died because of worsening in clinical feature. Other 4 patients had symptomatic grade 3 pneumonitis. These patients were hospitalized and treated with corticosteroids, and had clinical improvement. Two patients were treated after episode of pneumonitis without further lung complications. Other two patients had treatment discontinued. Immunotherapy doses used followed recommendations of their directions, excepted the dose used in case 1 that was based on a specific study that most participants had colorectal neoplasia and that did not have greater incidence of toxicities compared with other studies using a standard-dose.^(^ ^2^ ^)^ Coincidentally or not, the patient from case 1 had worsening in clinical feature and died because of complications in treatment. Another fact to consider is that delayed introduce of corticosteroids – due to fact that pneumonitis hypothesis was initially consider - might contributed to the unfavorable outcome.

Most observed radiologic pattern was ground-glass. Some patients had micronodules and consolidations. All patients were treated with antibiotics therapy and corticosteroids due to the impossibility to exclude associated pulmonary infection. No patient underwent biopsy, therefore, all were treated without pathological confirmation of pneumonitis, a common fact observed in other studies.^(^ ^1^ ^–^ ^5^ ^)^

Incidence of immunotherapy-induced pneumonitis in literature ranged from 2.7% to 5%.^(^ ^3^ ^,^ ^4^ ^)^ There was no difference in incidence of complication in comparison to different anti-PD1 such as nivolumab and pembrolizumab, however, we know that incidence increases when immunotherapies are combined – for example, nivolumab associated with anti-CTLA 4, and ipilimumab.^(^ ^3^ ^)^ Other important observation was the high incidence of any grade pneumonitis occurred among patients with lung as the primary site.^(^ ^4^ ^)^ Although this disease is uncommon, its death rate could reach up to 12%.^(^ ^3^ ^)^ Among radiological patterns observed, opacities in ground-glass were seen in all cases. This result corroborates with other studies in the literature that also show opacities in ground-glass as the most frequent radiological pattern.^(^ ^3^ ^)^ Treatment for pneumonitis is based on stopping all medication associated or not with oral or intravenous corticosteroid therapy, however, the stage of the disease needs to be considered.^(^ ^5^ ^)^ Immunotherapy treatment should be discontinued for grade 2 pneumonitis and definitively discontinued in case of recurrence grade 3, 4 and 4 pneumonitis (Table 1).^(^ ^6^ ^)^

**Table 1:** Toxicity grade and treatment^(^ ^6^ ^)^

Grade	Clinical-radiological findings	Management
1	Asymptomatic (only radiological changes)	Observation Consider discontinuation immunotherapy
2	Symptomatic (with limitation of instrumental daily changes)	Prednisone 1mg/kg/day (or similar) Discontinuation of immunotherapy drug
3	Symptomatic (with limitation of self-care) or hypothesis	Discontinue of immunotherapy drug
4	Symptomatic (with limitation of self-care) life threatening	Methylprednisolone EV 1-2mg/kg/day (or equivalent) Consider immunosuppressive drugs in the absence of improvement within 3 to 5 days
5	Death	

IV: intravenous.

## CONCLUSION

Early diagnosis of pneumonitis in patients undergoing immunotherapy treatment is extremely important because of great morbimortality of this complication and employment growth of this therapy in current oncology practice. This increase in the use has turned a previous rare event into a more frequent one.

This disease diagnosis is difficult because of the variability of clinical and radiological presentation. Medical team is responsible to identify most common symptoms such as cough and dyspnea to achieve early diagnosis and implement an effective treatment.
